# Supplementary material for: A Type I Restriction-Modification System Associated with Enterococcus faecium Subspecies Separation
Source: Appl Environ Microbiol. 2019 Jan 9;85(2):e02174-18. doi: 10.1128/AEM.02174-18 (PMC6328761; doi:10.1128/AEM.02174-18)
Supplement: Supplemental file 1 [file AEM.02174-18-s0001.pdf]

SUPPLEMENTAL MATERIAL

Table S1. *E. faecium* genomes used for conservation analysis.

Strain name	Clade	Category	Reference
EnGen0003	B	non-hospitalized individual	(1)
com12	B	non-hospitalized individual	(1)
EnGen0056	B	clinical isolate	(1)
EnGen0047	B	clinical isolate	(1)
1141733	B	clinical isolate	(1)
EnGen0038	B	clinical isolate	(1)
com12	B	non-hospitalized individual	(1)
EnGen0042	B	hospital feces	(1)
E980	B	non-hospitalized individual	(1)
EnGen0033	B	clinical isolate	(1)
EnGen0015	B	non-hospitalized individual	(1)
EnGen0028	B	other	(1)
LCT-EF90	B	other	(1)
EnGen0029	B	other	(1)
EnGen0026	B	clinical isolate	(1)
1231408	Rec	clinical isolate	(1)
EnGe0002	Rec	hospital surveillance	(1)
EnGen0013	A1	clinical isolate	(1)
EnGen0034	A1	clinical isolate	(1)
1230933	A1	clinical isolate	(1)
EnGen0046	A1	clinical isolate	(1)
U0317	A1	clinical isolate	(1)
E4452	A1	animal	(1)
EnGen0054	A1	clinical isolate	(1)
1231502	A1	clinical isolate	(1)
EnGen0049	A1	hospital surveillance	(1)
EnGen0045	A1	hospital surveillance	(1)
EnGen0016	A1	hospital unknown	(1)
EnGen0036	A1	clinical isolate	(1)
EnGen0030	A1	clinical isolate	(1)
E4453	A1	animal	(1)
EnGen0051	A1	hospital outbreak	(1)
EnGen0050	A1	clinical isolate	(1)
1231410	A1	clinical isolate	(1)
Aus0004	A1	clinical isolate	(1)
C68	A1	hospital outbreak	(1)
E1162	A1	clinical isolate	(1)

EnGen0057	A1	animal	(1)
EnGen0018	A2	animal	(1)
EnGen0031	A2	clinical isolate	(1)
EnGen0007	A2	animal	(1)
EnGen0017	A2	non-hospitalized individual	(1)
EnGen0025	A2	clinical isolate	(1)
EnGen0009	A2	animal	(1)
E1071	A2	hospital surveillance	(1)
EnGen0032	A2	animal	(1)
D344SRF	A2	other	(1)
TC6	A2	other	(1)
EnGen0011	A2	clinical isolate	(1)
E1636	A2	clinical isolate	(1)
EnGen0010	A2	animal	(1)
EnGen0048	A2	animal	(1)
EnGen0005	A2	animal	(1)
EnGen0022	A2	animal	(1)
EnGen0043	A2	animal	(1)
EnGen0027	A2	clinical isolate	(1)
EnGen0001	A2	animal	(1)
E1679	A2	hospital outbreak	(1)
EnGen0024	A2	clinical isolate	(1)
EnGen0020	A2	animal	(1)
EnGen0012	A2	clinical isolate	(1)
EnGen0044	A2	animal	(1)
1231501	A2	clinical isolate	(1)
EnGen0004	A2	clinical isolate	(1)
EnGen0052	A2	clinical isolate	(1)
EnGen0039	A2	other	(1)
EnGen0019	A2	animal	(1)
EnGen0040	A2	other	(1)
EnGen0008	A2	animal	(1)
EnGen0021	A2	hospital surveillance	(1)
E1039	A2	non-hospitalized individual	(1)
EnGen0014	A2	animal	(1)
EnGen0035	A2	clinical isolate	(1)

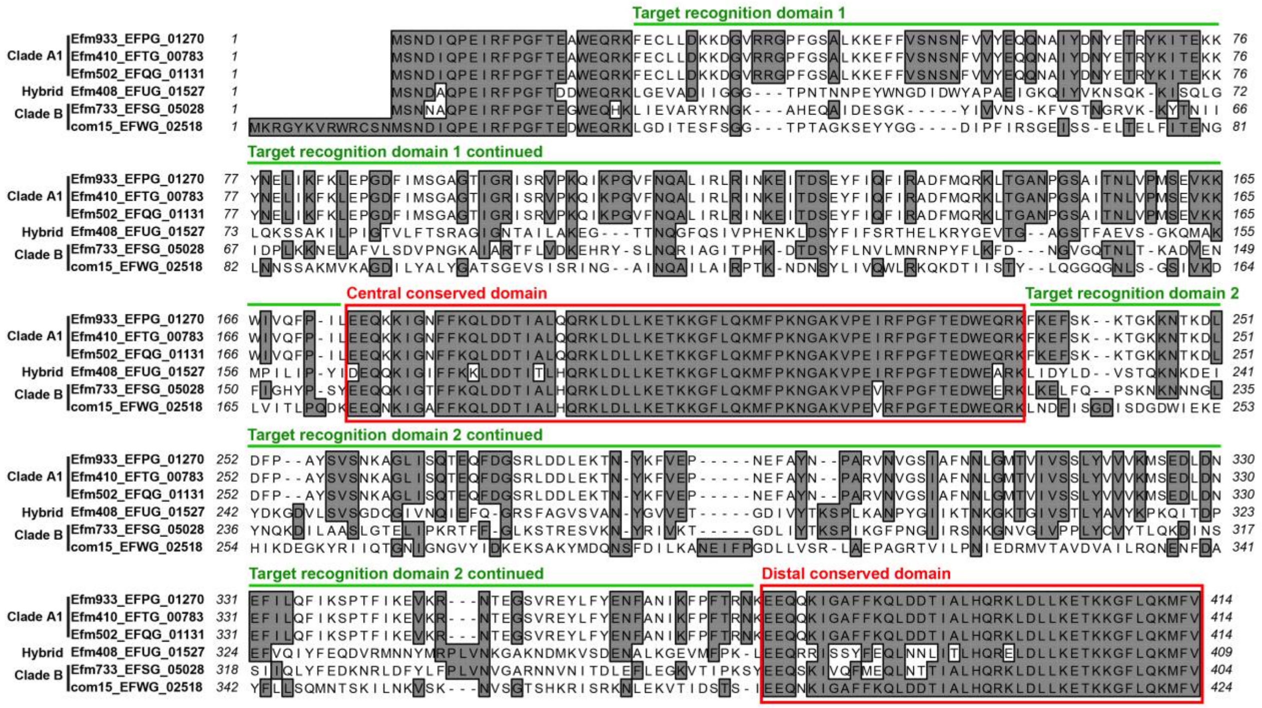

Figure S1. Multiple sequence alignment of S subunits. The protein sequences of predicted S subunits from 6 (out of 8) representative *E. faecium* genomes were aligned using MacVector. The multiple sequence alignment is shown here. Central and distal conserved domain was identified based on sequence homology and labeled in red. The target recognition domains were interpreted based on conserved domains as labeled in green.

		hsdS; EFQG_0113	*hsdR*; EFQG_0113	*hsdM*; EFQG_0113
Clade A1	EnGen0054			
Clade A1	EnGen0030			
Clade A1	EnGen0036			
Clade A1	EnGen0057		■	■
Clade A1	Aus0004		■	■
Clade A1	E4452		■	■
Clade A1	E4453		■	■
Clade A1	U0317	■	■	■
Clade A1	EnGen0046	■	■	■
Clade A1	1230933	■	■	■
Clade A1	1231410	■	■	■
Clade A1	1231502	■	■	■
Clade A1	C68	■	■	■
Clade A1	E1162	■	■	■
Clade A1	EnGen0013	■	■	■
Clade A1	EnGen0016	■	■	■
Clade A1	EnGen0034	■	■	■
Clade A1	EnGen0045	■	■	■
Clade A1	EnGen0049	■	■	■
Clade A1	EnGen0050	■	■	■
Clade A1	EnGen0051	■	■	■
Rec	EnGen0002	■	■	■
Rec	1231408		■	■
Clade A2	1231501			
Clade A2	D344SRF			
Clade A2	E1039		■	■
Clade A2	E1071			
Clade A2	E1636		■	■
Clade A2	E1679		■	■
Clade A2	EnGen0001		■	■
Clade A2	EnGen0004			
Clade A2	EnGen0005		■	■
Clade A2	EnGen0007		■	■
Clade A2	EnGen0008		■	■
Clade A2	EnGen0009		■	■
Clade A2	EnGen0010		■	■
Clade A2	EnGen0011		■	■
Clade A2	EnGen0012		■	■
Clade A2	EnGen0014			
Clade A2	EnGen0017			
Clade A2	EnGen0018		■	■
Clade A2	EnGen0019		■	■
Clade A2	EnGen0020		■	■
Clade A2	EnGen0021		■	■
Clade A2	EnGen0022			
Clade A2	EnGen0024		■	■
Clade A2	EnGen0025		■	■
Clade A2	EnGen0027		■	■
Clade A2	EnGen0031			
Clade A2	EnGen0032		■	■
Clade A2	EnGen0035		■	
Clade A2	EnGen0039		■	■
Clade A2	EnGen0040		■	■
Clade A2	EnGen0043		■	■
Clade A2	EnGen0044			
Clade A2	EnGen0048		■	■
Clade A2	EnGen0052			
Clade A2	TC6			
Clade B	1141733			
Clade B	com12			
Clade B	com15		■	■
Clade B	E980			
Clade B	EnGen0003		■	■
Clade B	EnGen0015			
Clade B	EnGen0026		■	■
Clade B	EnGen0028			
Clade B	EnGen0029		■	■
Clade B	EnGen0033			
Clade B	EnGen0038		■	■
Clade B	EnGen0042		■	■
Clade B	EnGen0047			
Clade B	EnGen0056		■	■
Clade B	LCT-EF90		■	■

a).

b).

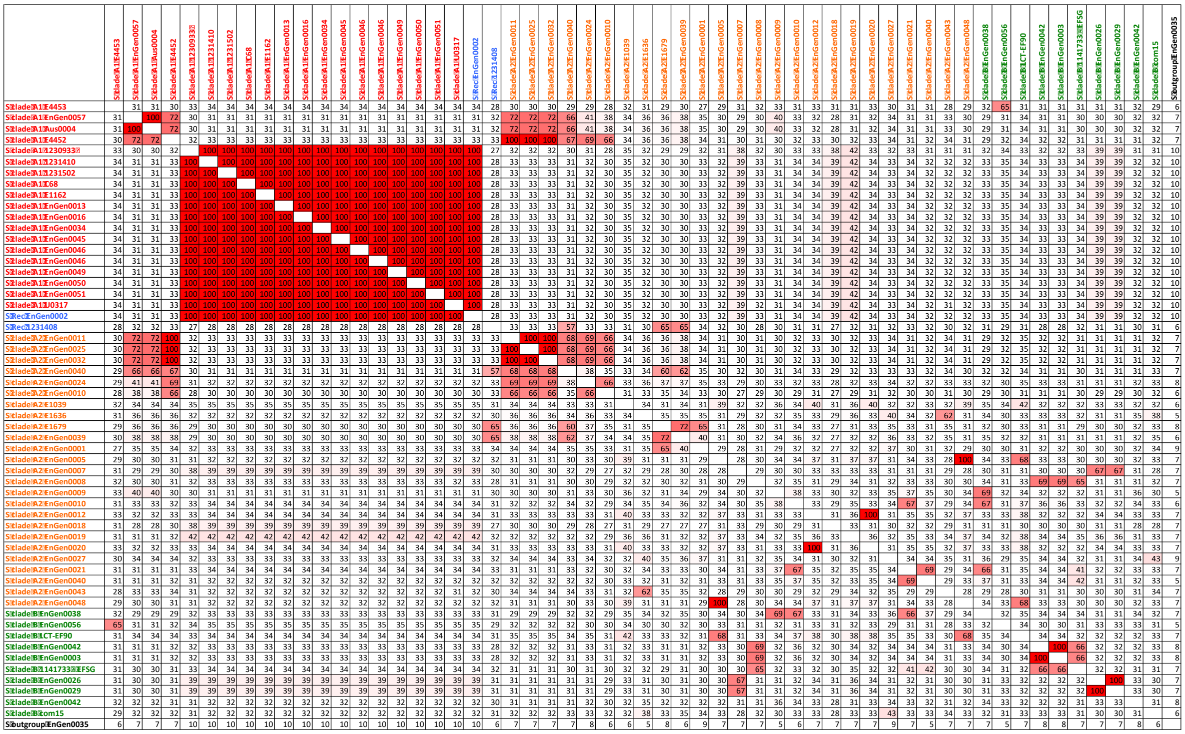

Figure S2. The distribution (a) and variation (b) of S subunits found in 52 (out of 73) *E. faecium* genomes. The nucleotide sequences of R, M and S subunits from Efa502I were used as reference and a collection of previously sequenced 73 *E. faecium* genomes were mapped to the reference using default settings (Medium-to-low sensitivity/Fast) in Geneious. The orthologs of R and M were identified in 52 and 51 genomes, respectively. The presence of R and M orthologs is shown in green (a). The S subunits were identified based on the neighboring R and M subunits. The protein sequences of all predicted S subunits were pairwise aligned using Geneious and the percent identity of each pair is shown and color-coded (b). White to red: low to high percent identities. EnGen0025 and EnGen0035 (names shown in black) were identified with R subunits but no S subunits were found in their vicinities, hence the hypothetical proteins next to the R subunits were extracted and used as out group in the pairwise alignment.

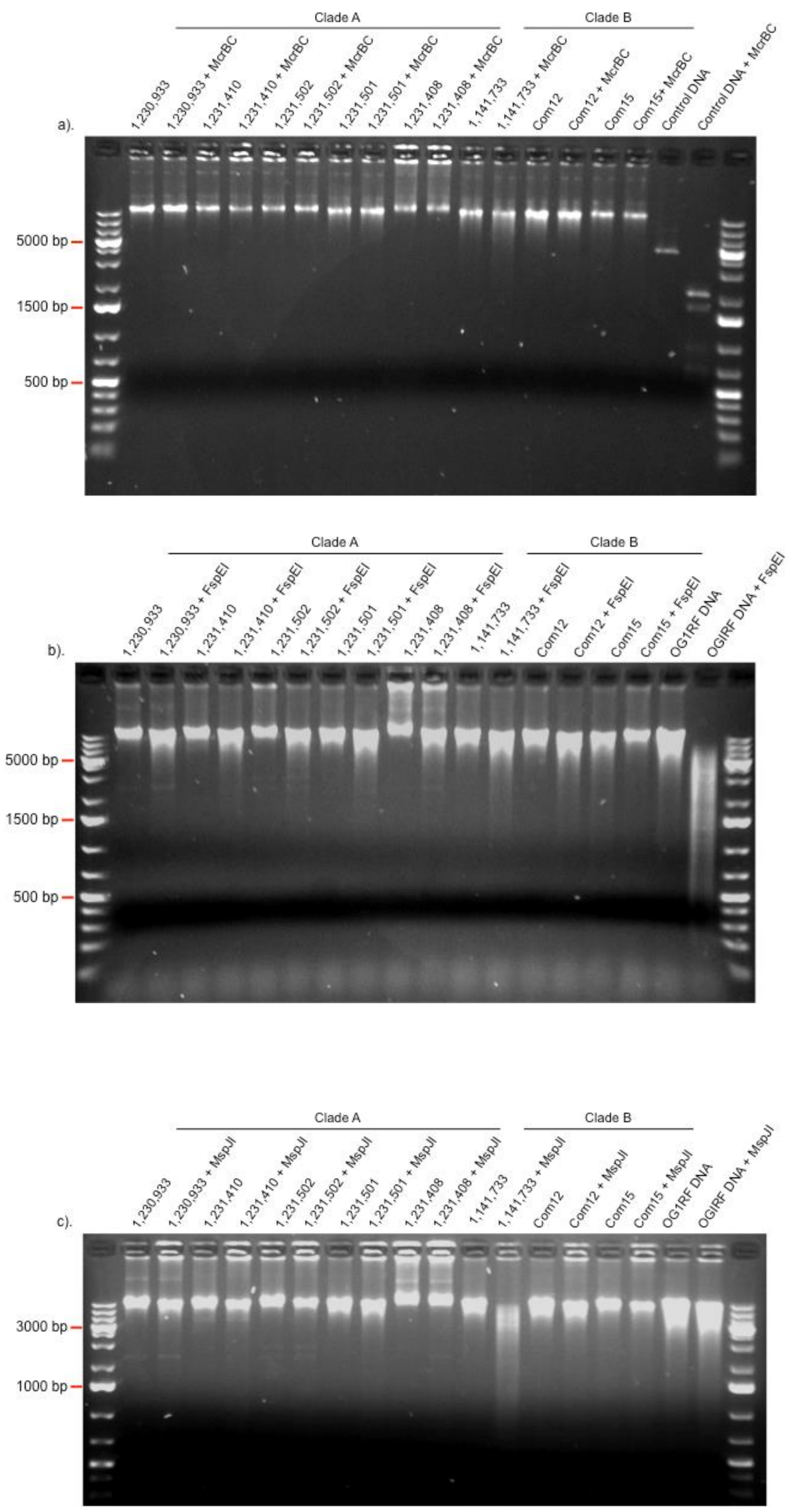

Figure S3. REase protection assays of representative *E. faecium* genomes. (a) McrBC, recognizing (G/A)^{m}C. Control DNA was provided by NEB and expected to be digested only in the presence of McrBC. (b) FspEI, recognizing fully methylated CpG or CHG (H=A/C/T). OG1RF

gDNA was used as positive control. OG1RF possesses m5C modification within G^{m5}C(A/T)GC motif hence its gDNA is expected to be digested by FspEI. (c) MspJI, recognizing ^{m}CNNR. OG1RF gDNA was used as negative control and no digestion was expected.

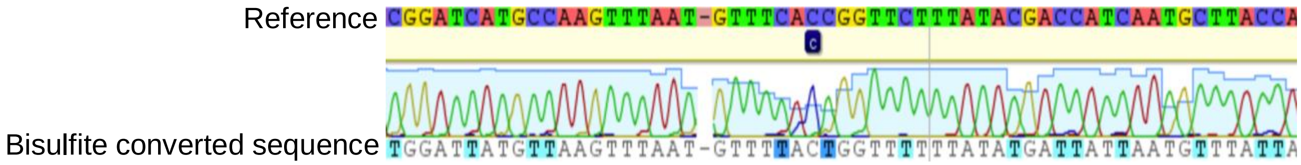

Figure S4. Targeted bisulfite sequencing of Efm733. Sequencing of Efm733 gDNA after bisulfite conversion. The marked C was protected from conversion, which indicates it has been modified by m5C methylation at 5’-R^{m}<u>C</u>CGGY-3’.

References

1. Lebreton F, van Schaik W, McGuire AM, Godfrey P, Griggs A, Mazumdar V, Corander J, Cheng L, Saif S, Young S, Zeng Q, Wortman J, Birren B, Willems RJ, Earl AM, Gilmore MS. 2013. Emergence of epidemic multidrug-resistant Enterococcus faecium from animal and commensal strains. MBio 4.
